# Supplementary material for: The Expression of Cellular Prion Protein, PrPC, Favors pTau Propagation and Blocks NMDAR Signaling in Primary Cortical Neurons
Source: Cells. 2023 Jan 11;12(2):283. doi: 10.3390/cells12020283 (PMC9856489; doi:10.3390/cells12020283)
Supplement: Supplementary file 1 [file cells-12-00283-s001.zip › cells-2074505-supplementary.pdf]

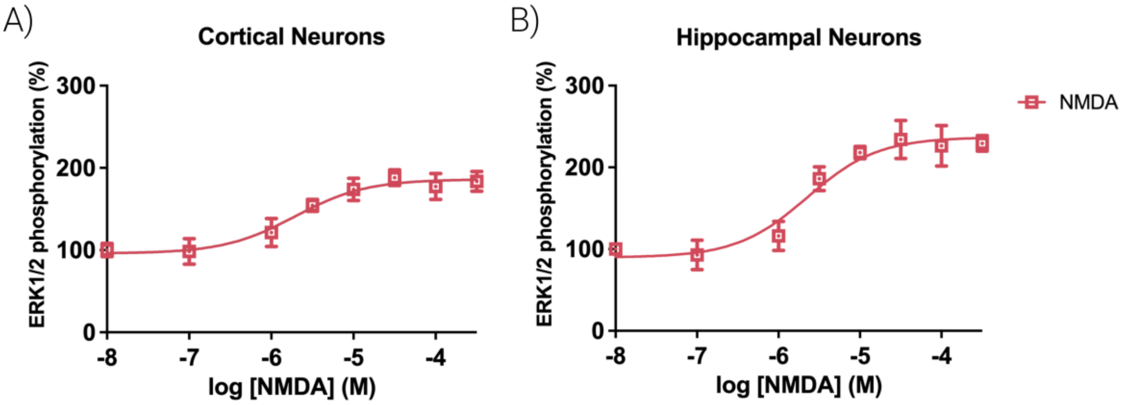

Figure S1. Dose–response curves on ERK1/2 phosphorylation upon the treatment with NMDA (red) of cortical neurons (Panel A) or hippocampal neurons (Panel B). ERK1/2 phosphorylation values are expressed as percentage mean with respect to basal levels ± S.E.M. of 5 independent experiments performed in triplicates.

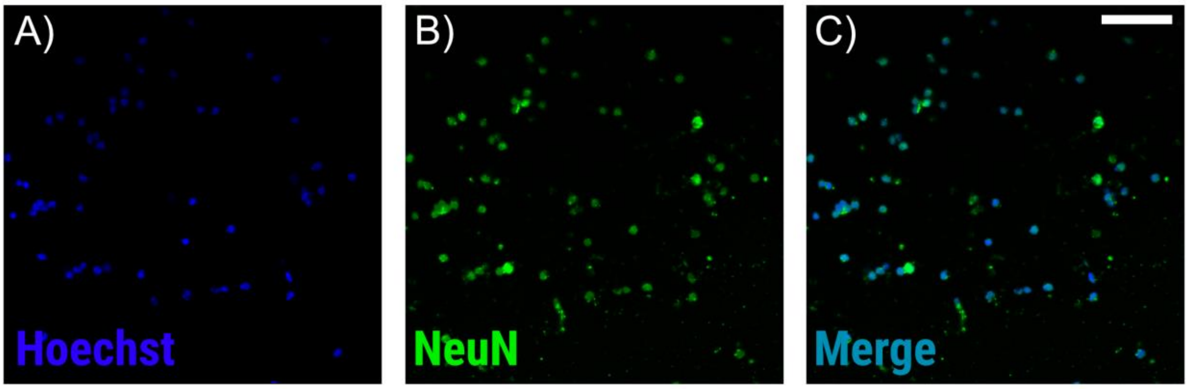

Figure S2. NeuN-488 neuronal marker immunolabeling for cortical neurons. A) Cell nuclei were stained with Hoechst (blue). B) Immunocytochemistry assays were performed to detect purity in primary cultures with the neuronal marker NeuN-Alexa Fluor® 488 rabbit monoclonal antibody (ab190195, green). C) Colocalization of both signals, shows major colocalization of the Neu-Alexa Fluor® 488 signal in the neurons nuclei.
